# Beneficial Effects of Neurotensin in Murine Model of Hapten-Induced Asthma

**DOI:** 10.3390/ijms20205025

**Published:** 2019-10-11

**Authors:** Ewelina Russjan, Katarzyna Kaczyńska

**Affiliations:** Department of Respiration Physiology, Mossakowski Medical Research Centre, Polish Academy of Sciences, 5 Pawińskiego Street, 02-106 Warsaw, Poland; erussjan@imdik.pan.pl

**Keywords:** neurotensin, asthma, inflammation

## Abstract

Neurotensin (NT) demonstrates ambiguous activity on inflammatory processes. The present study was undertaken to test the potential anti-inflammatory activity of NT in a murine model of non-atopic asthma and to establish the contribution of NTR1 receptors. Asthma was induced in BALB/c mice by skin sensitization with dinitrofluorobenzene followed by intratracheal hapten provocation. The mice were treated intraperitoneally with NT, SR 142948 (NTR1 receptor antagonist) + NT or NaCl. Twenty-four hours after the challenge, airway responsiveness to nebulized methacholine was measured. Bronchoalveolar lavage fluid (BALF) and lungs were collected for biochemical and immunohistological analysis. NT alleviated airway hyperreactivity and reduced the number of inflammatory cells in BALF. These beneficial effects were inhibited by pretreatment with the NTR1 antagonist. Additionally, NT reduced levels of IL-13 and TNF-α in BALF and IL-17A, IL12p40, RANTES, mouse mast cell protease and malondialdehyde in lung homogenates. SR 142948 reverted only a post-NT TNF-α decrease. NT exhibited anti-inflammatory activity in the hapten-induced asthma. Reduced leukocyte accumulation and airway hyperresponsiveness indicate that this beneficial NT action is mediated through NTR1 receptors. A lack of effect by the NTR1 blockade on mast cell activation, oxidative stress marker and pro-inflammatory cytokine production suggests that other pathways can be involved, which requires further research.

## 1. Introduction

Neurotensin (NT), regarded as a hormone, paracrine factor or neurotransmitter, regulates various physiological functions. It was first isolated from bovine hypothalamus by Carraway and Leeman in 1973 and named because of its neuronal localization and hypotensive activity [1]. In the brain, NT controls anterior pituitary hormone secretion [2], hypothermia [3] and antinociceptive activity [4]. In the periphery, NT is widely distributed in the gastrointestinal tract, where it influences gut motility. It has also been identified in other peripheral organs: lung, liver, heart, pancreas and spleen [5].

NT is an endogenous tridecapeptide (pGlu-Leu-Tyr-Glu-Asn-Lys-Pro-Arg-Arg-Pro-Tyr-Ile-Leu). The C-terminal part (precisely NT 8-13) is recognized by all known NT receptors and demonstrates activity similar to an entire NT molecule [6,7]. To date, four subtypes of cell surface receptors that can mediate NT responses have been described. The high-affinity NTR1 receptor, responsible for the most physiological effects of NT, is a member of the G protein-coupled receptors family, with seven transmembrane domains [8,9]. The G protein-linked second messenger system is also affected by the low-affinity NTR2 receptor, which presents 64% amino acid homology with NTR1. In contrast to NTR1, NTR2 is characterized by the capability to recognize levocabastine, a histamine H1 receptor antagonist. This sensitivity feature allows for the distinction between NTR1 and NTR2 [10]. However, the role of NTR2 in NT signaling remains controversial. Different results suggest that NT can be treated as an agonist, inverse agonist or antagonist at this binding site [11]. Some similarities have been described between mosaic protein NTR3 and the last identified NTR4 receptor. However, their role in the physiological properties of NT is unclear [12,13].

Neurotensin plays a part as an important regulator of immune cells: lymphocytes, mastocytes and macrophages, as well as vascular, epithelial and connective tissue cells [14,15]. Similarly to other neuropeptides, NT can act as a link between the immune and nervous systems. Neurogenic stimulation is involved in the processes of inflammation, proliferation, cytokine and growth factor production [16] and this neuroimmune interaction can be observed in asthma [17]. Asthmatic airway hyperreactivity is modulated by the neurons innervating lungs [18,19]. NT receptors have been synthesized and transported within a subpopulation of afferent and efferent components of the vagus nerve [20]. Therefore, a possibility exists that, in asthma, NT might exert its effects via the modulation of both inflammatory cells and lung nerves.

Despite considerable advances in understanding NT properties and its function in physiological and pathological phenomena, its exact role in the inflammation process still remains controversial. There are several studies, demonstrating contradicting results, which point to either the pro- or anti-inflammatory activity of NT. As an example, it has been demonstrated that NT promotes an acute inflammatory response in the experimental model of colon inflammation [21], while in another study, NT augmented the healing process of colonic mucosa in the chronic phase of disease [22].

The controversies on NT involvement in the inflammatory processes and the possibility of modulatory action on lung nerves prompted us to investigate its potential anti-inflammatory activity in a murine model of non-atopic asthma induced by a low molecular compound (hapten), specifically dinitrofluorobenzene (DNFB). A simultaneous purpose is establishing the contribution of the NTR1 receptor via pretreatment with the nonpeptide NT antagonist SR 142948.

## 2. Results

### 2.1. Airway Hyperresponsiveness (AHR)

**Figure 1:**
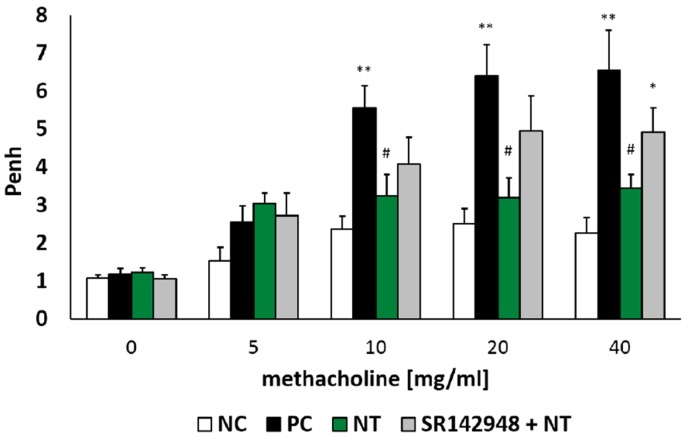
Airway responsiveness (Penh) to increasing concentrations of methacholine (5, 10, 20, 40 mg/mL). Effect of treatment with Neurotensin (NT) alone and NT pretreated with SR 142948 in dinitrofluorobenzene (DNFB)-sensitized/ dinitrobenzene sulphonic acid (DNS)-challenged mice. Comparison to DNFB-sensitized/DNS-challenged group (positive control; PC) and vehicle-sensitized/DNS-challenged group (negative control; NC) treated with NaCl. The results are presented as means ± SEM; n = 6–7 mice per group. * *p* < 0.05, ** *p* < 0.01 compared with the NC group, ^#^ *p* < 0.05 compared with the PC group.

There was no significant difference between all tested groups after NaCl and the lowest dose of methacholine (5 mg/mL) nebulization. However, an intratracheal dinitrobenzene sulphonic acid (DNS) challenge in DNFB-sensitized mice increased airway hyperresponsiveness to methacholine in doses of 10, 20 and 40 mg/mL, which was reflected in increased Penh (Figure 1). Intraperitoneal injection of NT after the hapten challenge significantly reduced Penh compared to the positive control group (PC). Administration of the NT antagonist SR 142948 limited the ameliorative activity of NT on airway hyperresponsiveness (Figure 1). Although there was no meaningful difference in Penh values between NT and SR 142948 + NT treated groups (*p* = 0.3, 0.09 and 0.15 for the three highest methacholine doses), antagonist pretreatment counteracted NT-induced AHR reduction in comparison to the PC group (*p* = 0.07, 0.11 and 0.15 for 10, 20 and 40 mg/mL of methacholine).

### 2.2. Leukocyte Accumulation in BALF and Lung Tissue

**Figure 2:**
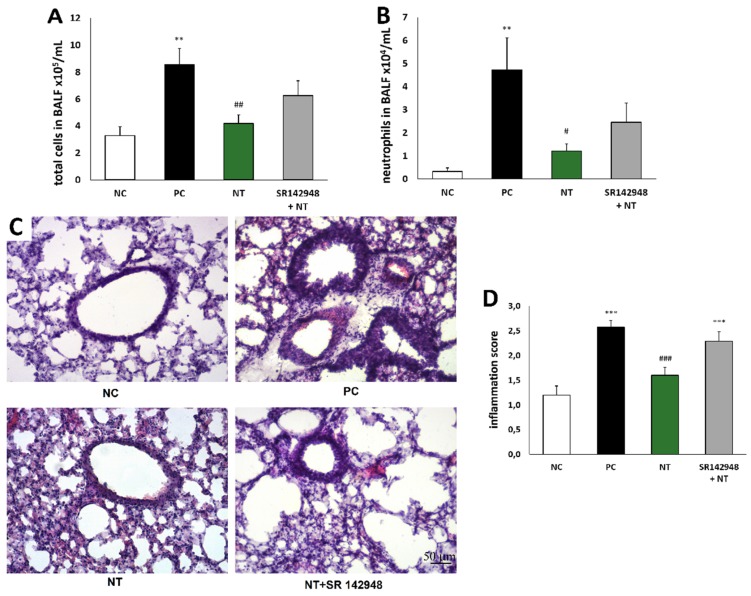
Reduction of the total number of inflammatory cells (**A**) and neutrophils (**B**) in bronchoalveolar lavage fluid (BALF) and effect of NT on lung tissue inflammatory cell infiltration; hematoxylin- and eosin-stained lung sections (**C**) and inflammation score (**D**); see Materials and Methods 4.9. Effect of treatment with NT alone and NT pretreated with SR 142948 in comparison to DNFB-sensitized/DNS-challenged group (positive control; PC) and vehicle-sensitized/DNS-challenged group (negative control; NC) treated with NaCl. The pictures were made at magnification × 10. The results are presented as means ± SEM (n = 7–8 in BALF and n = 4 in histology study). ** *p* < 0.01, *** *p* < 0.001 compared with the NC group, ^#^ *p* < 0.05, ^##^ *p* < 0.01, ^###^ *p* < 0.001 compared with the PC group.

The cell influx in the airway lumen during the course of inflammation consisted primarily of macrophages and neutrophils, and, to a lesser degree, lymphocytes. The higher percentage of neutrophils is a hallmark of non-atopic asthma. DNFB-sensitized/DNS-challenged mice (PC) demonstrated an increased number of total inflammatory cells and neutrophils compared to a vehicle-sensitized/DNS-challenged group (NC) (Figure 2A,B). The administration of NT significantly reduced leukocyte accumulation in BALF. The beneficial effect of NT was reduced by pretreatment with SR 142948 (Figure 2A,B). A histological evaluation showed similar changes—apparent peribronchial leukocyte accumulation in the PC group (Figure 2C), which was reflected by an increased inflammation score (Figure 2D). NT treatment significantly attenuated the inflammation score, while pretreatment with the NTR1 antagonist eliminated this effect (Figure 2D).

### 2.3. Pro-Inflammatory Cytokine Production

**Figure 3:**
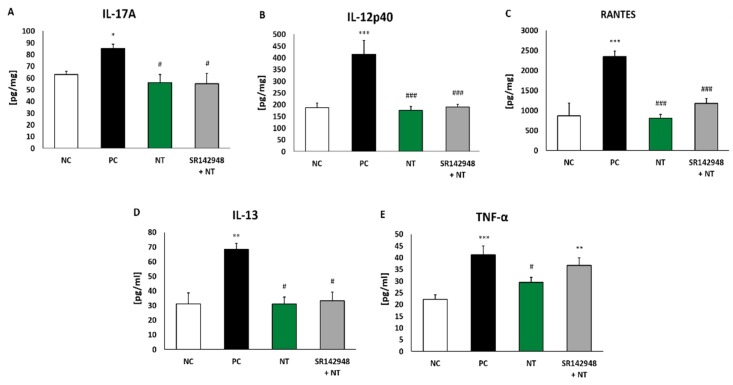
Pro-inflammatory cytokine concentrations in lung tissue homogenates: IL-17A (**A**), IL-12p40 (**B**), RANTES (**C**) and in BALF: IL-13 (**D**) and TNF-α (**E**) after treatment with NT alone and NT pretreated with SR 142948. Comparison to DNFB-sensitized/DNS-challenged group (positive control; PC) and vehicle-sensitized/DNS-challenged group (negative control; NC) treated with NaCl. The results are presented as means ± SEM; n = 5–7 mice per group. * *p* < 0.05, ** *p* < 0.01, *** *p* < 0.001 compared with the NC group; ^#^ *p* < 0.05, ^###^ *p* < 0.001 compared with the PC group.

An increased concentration of IL-17A, IL-12p40 and RANTES in lung tissue homogenates and IL-13 and TNF-α in BALF was found in the PC group (Figure 3A–E). DNFB-sensitized/DNS-challenged mice treated with NT were characterized as having a much lower level of cytokines compared to the group treated with NaCl. Pretreatment with SR 142948 did not antagonize the anti-inflammatory activity of NT on interleukin production (Figure 3A–E). The only exception was the concentration of TNF-α, which remained enhanced after pretreatment with the NT antagonist (Figure 3E).

### 2.4. The Level of Mouse Mast Cell Protease (MCPT 1)

**Figure 4:**
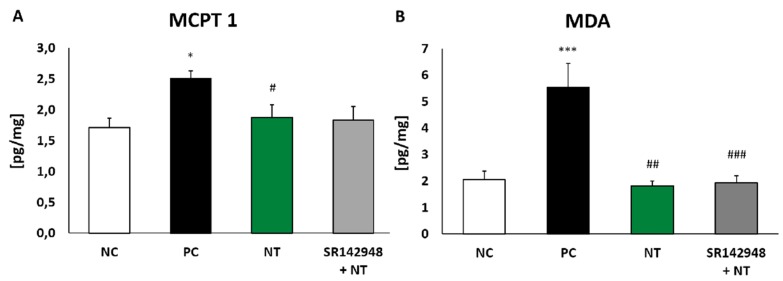
Mouse mast cell protease-1 (MCPT 1) (**A**) and malondialdehyde (MDA) (**B**) concentration in lung tissue homogenates after treatment with NT alone and NT pretreated with SR 142948. Comparison to DNFB-sensitized/DNS-challenged group (positive control; PC) and vehicle-sensitized/DNS-challenged group (negative control; NC) treated with NaCl. The results are presented as means ± SEM; n = 4–7 mice per group. * *p* < 0.05, *** *p* < 0.001 compared with the NC group; ^#^ *p* < 0.0, ^##^ *p* < 0.01, ^###^ *p* < 0.001 compared with the PC group.

The injection of DNS into the trachea of DNFB-sensitized mice significantly increased MCPT 1 concentration compared to the same in vehicle-sensitized animals. The administration of NT alone and NT pretreatment with SR 142948 reduced levels of protease in lung tissue homogenates. However, a significant difference was only obtained in the group without the application of an antagonist (Figure 4A).

### 2.5. Oxidative Stress Marker Production

Malondialdehyde (MDA) concentration in lung homogenates of DNFB-sensitized/DNS-challenged mice was almost three times that of the negative control (Figure 4B). NT treatment completely inhibited oxidative stress marker production, while the administration of the SR 142948 antagonist had no influence on NT activity.

## 3. Discussion

Although NT was isolated and described almost fifty years ago, its role in the regulation of an inflammatory response is not completely elucidated. Some studies indicate the pro-inflammatory activity of NT. It has been evidenced that plasma and intraperitoneal concentrations of NT are elevated in the murine model of sepsis, whereas the administration of the NT antagonist or using NT-deficient mice resulted in a decreased mortality rate [23]. The latest study suggested that the plasma level of the stable precursor of NT may be a biomarker of visceral adipose tissue inflammation [24]. The expression of NT and NTR1 was upregulated in animal models of colitis, suggesting that in the gastrointestinal system NT is involved in pro-inflammatory signaling [25].

On the other hand, there is evidence pointing to an ameliorative effect of NT in the development of inflammatory responses in the colon, confirmed by the repair and regeneration of colonic mucosa [22,26]. NT signaling was upregulated in experimental colitis as a part of the adaptive mechanism involved in the healing process, while the administration of the NT antagonist increased the severity of mucosal injuries. The beneficial influence of NT on wound repair has been associated with epithelial cell migration mediated through COX-2 activation and PGE_2_ production [27]. In another study the neuropeptide stimulated intestinal mucosa regeneration and exhibited anti-inflammatory activity through the reduction of IL-6 and TNF-α levels in serum, and myeloperoxidase, malondialdehyde and caspase-3 in colonic tissue [28]. The healing properties of NT have been confirmed in a murine model of diabetes, where collagen matrices, loaded with NT-enhanced wound healing, decreased the expression of TNF-α and IL-1β and reduced the infiltration of inflammatory cells into the wound [29]. Furthermore, a crucial role of NT in the wound healing process was evidenced by a decrease in the pro-inflammatory features of skin dendritic cells and fibroblasts and an increase in the epidermal growth factor expression [30,31].

The discrepant data on NT’s role in inflammation and the possibility of modulatory action on lung nerves prompted us to investigate its potential in a murine model of non-atopic asthma, in which an inflammatory reaction has been elicited by dermal sensitization with DNFB and an intratracheal challenge with cognate compound DNS, as was previously described by van der Kleij [32].

In our study, NT administration markedly reduced airway responsiveness to methacholine provocation and the effect was blocked by pretreatment with the NTR1 antagonist. In fact, widespread NT in components of the central and peripheral nervous systems has been postulated to regulate breathing in the physiological state and in the disease [33]. The neuropeptide and its receptors have been identified in the airway mucosa and lungs [34], and in the presynaptic cholinergic terminals and post-synaptic smooth muscles of the bronchi [35], indicating potential sites of NT action and the possibility of the modulation of airway hyperresponsiveness.

Yet, an in vitro study on guinea pig and rat tracheal preparations demonstrated the contractile properties of NT on airway smooth muscle [35,36]. Contrarily another subsequent finding showed that NT inhibited cholinergic and noncholinergic contractions of guinea pig bronchial rings stimulated by an electrical field. The effect was reversed after treatment with a selective NTR1 receptor antagonist, which corresponds to our AHR results [37]. We cannot rule out that reduced airway hyperresponsiveness induced by NT was the consequence of attenuated inflammation reflected in the decreased inflammatory cell influx in BALF, blocked by the NTR1 antagonist, as well. We used SR 142948, which is not as selective an antagonist as SR 48692 but presents approximately ten times the affinity for NTR1 than it does for NTR2 [38]. SR 48692, although characterized by high selectivity, failed to block many physiological effects of NT, such as hypothermia and analgesia [39,40]. Its newer potent antagonist, SR 142948, has the ability to antagonize a wider spectrum of NT-mediated effects [41,42].

The contribution of NTR2 in these phenomena remains unclear when taking into consideration the fact that several cell line studies indicate that both NT antagonists, SR 48962 and SR 142948 may activate, instead of block, NTR2 [43]. The ability to examine NTR2 involvement in the inflammatory process is limited due to the lack of a selective NTR2 antagonist. Levocabastine, which is recognized by NTR2, exhibits species-dependent activity—it acts as an agonist in mice and as an antagonist in humans [44].

The alleviating properties of NT have been further confirmed in our study in cytokine assays, where NT diminished the concentration of IL-12p40, RANTES and IL-17A in lung homogenates, and IL-13 and TNF-α in BALF. Many interleukins and chemokines have been described as being involved in the development of asthma. IL-12p40, which is produced by activated monocytes, neutrophils, macrophages and dendritic cells, acts as an inducer of Th1 cell differentiation [45]. Yet, anti-IL-12 treatment during the airway challenge was able to reduce the main symptoms of ovalbumin-induced asthma, suggesting that IL-12 plays a pro-inflammatory role in the effector phase of allergic airway inflammation [46]. Pro-inflammatory activity has also been demonstrated in chemokine RANTES, which is considered to be a chemoattractant for eosinophils, monocytes, macrophages and T lymphocytes. An elevated level of RANTES mRNA was observed in the respiratory tract of patients suffering from mild asthma [47]. RANTES concentration was also markedly higher in the exhaled breath condensate of asthmatics, particularly in patients with unstable asthma [48].

Another interleukin playing an important role in pulmonary inflammation is IL-17A, which is secreted by distinctive T cells of the Th17 subtype and belongs to a larger IL-17 cytokine family [49]. IL-17A is expressed in the BALF, sputum and bronchial biopsies of asthmatic patients and is closely linked to neutrophilic influx into the airways [50]. Neutrophil-mediated inflammation is connected with the stimulating activity of IL-17A, which induces the release of the potent neutrophil chemotactic factor CXCL8 from airway smooth muscle cells and airway epithelial cells, and in this way mobilizes neutrophil recruitment [51,52]. These results are in line with the present study, where an increased number of neutrophils in the BALF of DNFB-sensitized/DNS-challenged mice was accompanied by an elevated concentration of IL-17A in lung tissue homogenates.

Another cytokine whose concentration in BALF was reduced by NT treatment is IL-13, regarded as one of the most crucial cytokines in the initiation and exacerbation of asthma in humans [53]. This Th2 cytokine plays an important role in allergic inflammation, yet acts probably via the pathway independent of immunoglobulin E and eosinophils. IL-13 gene polymorphism might have an impact on susceptibility to the disease [54].

It is noteworthy that pretreatment with the NT antagonist SR 142948 did not inhibit the effect of NT on the majority of cytokines examined, suggesting that pathways for a receptor other than NTR1 might be involved. The exception was TNF-α, which was attenuated by NT, and the effect was reversed after the NTR1 blockade. Similarly to our study, NT reduced the level of TNF-α in the serum of rats with experimentally induced colitis [28]. It has been previously demonstrated that the application of TNF-α to healthy and asthmatic subjects led to airway hyperresponsiveness and neutrophil infiltration [55,56]. Increased levels of TNF-α were confirmed in bronchoalveolar lavage in the same DNFB-induced model of non-allergic asthma [32]. The main source of TNF-α in asthmatic lungs is mast cells, contributing to the development of late AHR [57]. The mast cells are known to store a large number of different mediators, including TNF-α, that might be released at the same time as other preformed granule contents, such as histamine, tryptase or chymase [58]. One of those compounds is MCPT 1, classified as a β-chymase and predominantly produced by mucosal mast cells [59]. In the present study, the DNS challenge in DNFB-sensitized mice increased the level of MCPT 1 in lung tissue and the effect was markedly reduced after NT administration. The key role of mast cells was previously established in a DNFB-induced model for non-atopic asthma [60].

We also demonstrated that NT significantly reduced the level of MDA, which corresponds to results obtained by Akcan et al. [28]. MDA, regarded as a marker of oxidative stress reaction, is formed as a consequence of the peroxidation of polyunsaturated fatty acids, which form the lipids in the cell membrane. The concentration of MDA is increased both in the exhaled breath condensate and the serum of asthmatic patients [61]. This is in line with animal studies—MDA was elevated in murine lung tissue homogenates in a model of ovalbumin-induced allergic asthma [62], as well as in occupational asthma in rats provoked by toluene diisocyanate [63], which supports the hypothesis that an antioxidative–prooxidative imbalance is one of the characteristic features of the inflammatory process in lungs.

Summarizing, the present study demonstrated NT potency in modulating the inflammatory response and airway hyperreactivity in a non-atopic asthma model. The effect of NT on leukocyte accumulation, airway hyperreactivity and the TNF-α level was mediated through NTR1 receptors. On the contrary, the lack of impact of SR 142948 on the beneficial effect of NT on pro-inflammatory cytokine production and oxidative stress indicates that another receptor pathway might be involved in the reaction. Further experiments investigating the detailed mechanism of NT ameliorative activity are required.

## 4. Materials and Methods

### 4.1. Drugs and Reagents

Neurotensin (NT) and neurotensin antagonist SR 142948 were purchased from Tocris Bioscience (Bristol, UK). 1-fluoro-2,4-dinitrobenzene (DNFB), dinitrobenzene sulfphonic acid (DNS), TX-100 and protease inhibitor cocktail were obtained from Sigma-Aldrich (Poznań, Poland). Pentobarbital sodium, ketamine and xylazine were acquired from Biowet (Puławy, Poland).

### 4.2. Animals and Hapten-Induced Experimental Asthma Model

Male BALB/c mice 7–8 weeks of age were obtained from the Animal House of Mossakowski Medical Research Centre, Polish Academy of Sciences. All animal experiments were conducted in accordance with the guidelines approved (15 April 2014) by the IV Warsaw Ethics Commission for the Care and Use of Laboratory Animals (permit number: 15/2014).

On day 0, mice were sensitized dermally with 0.5% DNFB (dissolved in acetone and olive oil (ratio 4:1) or vehicle control onto shaved thorax (50 μL) and on paws (50 μL on four paws). On day 1, DNFB/vehicle was only applied to the thorax. On day 5, mice were challenged with 50 μL of 0.6% DNS (dissolvable in water cognate hapten of DNFB) by intratracheal application. All procedures were performed under light anesthesia using ketamine (70 mg/kg IM) and xylazine (10 mg/kg IM). Twenty-four hours later, the airway responsiveness to methacholine exposure was measured. After that, the mice were sacrificed by intraperitoneal injection of pentobarbital sodium (150–200 mg/kg). Bronchoalveolar lavage fluid (BALF) and lung tissue were collected in order to perform further assays.

### 4.3. Experimental Design

The study was performed in four experimental groups (*n* = 8 in each group):

I vehicle-sensitized, DNS-challenged mice treated with physiological saline (NaCl) as the negative control (NC);
II DNFB-sensitized, DNS-challenged mice treated with NaCl as the positive control (PC);
III DNFB-sensitized, DNS-challenged mice treated with neurotensin alone (NT);
IV DNFB-sensitized, DNS-challenged mice pretreated with SR 142948 and treated with NT (SR 142948 + NT).

The doses of NT and SR 142948 were 7.4 μmol/kg and 2.9 μmol/kg, respectively, and were taken from previous studies [42,64]. Compounds were dissolved in NaCl (0.9%). All groups were treated twice—two and eight hours after DNS challenge by the intraperitoneal administration of equivalent volumes of 100 μL of NaCl (I, II), NT (III) or SR 142948 + NT (IV). The interval between the administration of the antagonist and the NT in the last group was thirty minutes.

### 4.4. Airway Hyperresponsiveness

The measurements of bronchial hyperresponsiveness were performed in the murine whole body plethysmograph chamber (Buxco Electronics, Inc., Wilmington, NC, USA) twenty-four hours after the intratracheal DNS challenge. The animals were placed in the chamber and after ten minutes of adaptation they were exposed to the NaCl aerosol control. Then, every twenty minutes, increasing concentrations of methacholine (5, 10, 20 and 40 mg/mL) were administered. Airway hyperresponsiveness was reflected by increasing values of enhanced pause (Penh), which were recorded for three minutes after each nebulization.

### 4.5. Leukocyte Accumulation in BALF

After sacrificing each mouse, a cannula was placed into the trachea and four aliquots of the phosphate buffered saline (PBS) solution were inserted and withdrawn (4 × 1 mL). The bronchoalveolar lavages collected were centrifuged (1500 rpm, 10 min) in order to separate the BALF cells from the supernatant. The cell pellet was then re-suspended in 150 μL of PBS. Türk reagent (100 μL) was added to 50 μL of the suspension and the total number of inflammatory cells was determined using a Bürker counting chamber. The remaining cell suspension was centrifuged with a cytocentrifuge (700 rpm for 10 min, Thermo Shandon, Cambridge, UK). The microscopic slides obtained were left to dry and then stained with a fast staining kit based on eosin and azure solutions (Hemastain, Analab, Warsaw, Poland). The leukocyte differentiation was determined using a light microscope and by counting the number of mononuclear cells and neutrophils per 500 consecutive cells in each sample. Results are expressed as the number of cells per 1 mL of BALF.

### 4.6. Pro-Inflammatory Cytokine Production

The first 1 mL of BALF, containing the cocktail of protease inhibitors, was centrifuged (1500 rpm, 10 min) and the supernatant obtained was collected and stored at −80 °C. After collecting the BALF, the lungs were isolated and also transferred to −80 °C. For further testing, the lungs were homogenized in liquid nitrogen and suspended in 500 μL of PBS, with the addition of a protease inhibitor cocktail and 1% Triton X-100. The sample was then centrifuged (14,000 rpm, 10 min, 4 °C) and the supernatant was used to analyze cytokine levels. Measurements of IL-13, TNF-α in BALF and IL-17A, IL-12p40 and RANTES in lung tissue homogenates were taken using the Bio-Plex Pro Mouse Cytokine panel (Bio-Rad, Warsaw, Poland) with the Bio-Plex 200 platform (Luminex, Bio-Rad, Warsaw, Poland).

### 4.7. The Level of Mouse Mast Cell Protease (MCPT 1)

The level of mouse mast cell protease (MCPT 1), a type of serine protease (β-chymase) stored and secreted in a tissue-specific manner by mucosal mast cells, was determined in lung tissue homogenates using the Mouse MCP-1 ELISA Ready-SET kit (eBioscience, San Diego, CA, USA) according to the manufacturer’s guidelines.

### 4.8. Oxidative Stress Marker Production

Malondialdehyde (MDA) is a product of lipid peroxidation and a marker of oxidative stress. Determination of MDA concentration in the lung tissue homogenates was performed by the ELISA enzyme assay using a commercially available analysis kit (OxiSelect MDA, Cell Biolabs, Inc., San Diego, CA, USA).

### 4.9. Lung Histology

Sections of lung tissue, 20 μm in thickness, were stained with eosin and hematoxylin (Merck Millipore, Poland) and examined under a light microscope (Nikon, Japan). To assess the severity of immune cell infiltration, peribronchial and perivascular cell counts were performed based on the four-point scoring system (from 0 to 3). Briefly, the four-point scoring system described by Tourney et al. [65] was: no inflammation detectable (0), occasional cuffing with inflammatory cells (1), most bronchi or vessels surrounded by a thin ring (one to five cells) of inflammatory cells (2), most bronchi or vessels surrounded by a thick layer (more than five cells) of inflammatory cells (3).

### 4.10. Statistical Analysis

The results are presented as mean ± standard error of mean (SEM). The concentration of MCPT 1, MDA and pro-inflammatory cytokines in lung tissue homogenates is expressed per mg of total protein in the sample. Statistical analysis was performed using a one-way ANOVA test with a Newman–Keuls post-hoc test. The results were considered significant when the significance level of *p* was less than 0.05. Analyses were performed using the STATISTICA software 12 (StatSoft, Kraków, Poland).

## References

[1] Carraway R., Leeman S.E. (1973). The isolation of a new hypotensive peptide, neurotensin, from bovine hypothalami. J. Biol. Chem..

[2] Bello A.R., Reyes R., Hernández G., Negrín I., González M., Tramu G., Alonso R. (2004). Developmental expression of neurotensin in thyrotropes and gonadotropes of male and female rats. Neuroendocrinology.

[3] Bissette G., Nemeroff C.B., Loosen P.T., Prange A.J., Lipton M.A. (1976). Hypothermia and intolerance to cold induced by intracisternal administration of the hypothalamic peptide neurotensin. Nature.

[4] Clineschmidt B.V., McGuffin J.C. (1977). Neurotensin administered intracisternally inhibits responsiveness of mice to noxious stimuli. Eur. J. Pharmacol..

[5] Boules M., Li Z., Smith K., Fredrickson P., Richelson E. (2013). Diverse roles of neurotensin agonists in the central nervous system. Front. Endocrinol. (Lausanne).

[6] Lambert P.D., Gross R., Nemeroff C.B., Kilts C.D. (1995). Anatomy and mechanisms of neurotensin-dopamine interactions in the central nervous system. Ann. N.Y. Acad. Sci..

[7] Vincent J.P., Mazella J., Kitabgi P. (1999). Neurotensin and neurotensin receptors. Trends Pharmacol. Sci..

[8] Tanaka K., Masu M., Nakanishi S. (1990). Structure and functional expression of the cloned rat neurotensin receptor. Neuron.

[9] Vita N., Laurent P., Lefort S., Chalon P., Dumont X., Kaghad M., Gully D., Le Fur G., Ferrara P., Caput D. (1993). Cloning and expression of a complementary DNA encoding a high affinity human neurotensin receptor. FEBS Lett..

[10] Chalon P., Vita N., Kaghad M., Guillemot M., Bonnin J., Delpech B., Le Fur G., Ferrara P., Caput D. (1996). Molecular cloning of a levocabastine-sensitive neurotensin binding site. FEBS Lett..

[11] Kleczkowska P., Lipkowski A.W. (2013). Neurotensin and neurotensin receptors: Characteristic, structure-activity relationship and pain modulation—A review. Eur. J. Pharmacol..

[12] Mazella J., Zsürger N., Navarro V., Chabry J., Kaghad M., Caput D., Ferrara P., Vita N., Gully D., Maffrand J.P. (1998). The 100-kDa neurotensin receptor is gp95/sortilin, a non-G-protein-coupled receptor. J. Biol. Chem..

[13] Jacobsen L., Madsen P., Jacobsen C., Nielsen M.S., Gliemann J., Petersen C.M. (2001). Activation and functional characterization of the mosaic receptor SorLA/LR11. J. Biol. Chem..

[14] Moura L.I., Cruz M.T., Carvalho E. (2014). The effect of neurotensin in human keratinocytes-implication on impaired wound healing in diabetes. Exp. Biol. Med. (Maywood).

[15] Katsanos G.S., Anogianaki A., Castellani M.L., Ciampoli C., De Amicis D., Orso C., Pollice R., Vecchiet J., Tetè S., Salini V. (2008). Biology of neurotensin: Revisited study. Int. J. Immunopathol. Pharmacol..

[16] Schäffer M., Beiter T., Becker H.D., Hunt T.K. (1998). Neuropeptides: Mediators of inflammation and tissue repair?. Arch. Surg..

[17] Voisin T., Bouvier A., Chiu I.M. (2017). Neuro-immune interactions in allergic diseases: Novel targets for therapeutics. Int. Immunol..

[18] Barnes P.J. (1986). Asthma as an axon reflex. Lancet.

[19] Tränkner D., Hahne N., Sugino K., Hoon M.A., Zuker C. (2014). Population of sensory neurons essential for asthmatic hyperreactivity of inflamed airways. Proc. Natl. Acad. Sci. USA..

[20] Kessler J.P., Beaudet A.J. (1989). Association of neurotensin binding sites with sensory and visceromotor components of the vagus nerve. J. Neurosci..

[21] Castagliuolo I., Wang C.C., Valenick L., Pasha A., Nikulasson S., Carraway R.E., Pothoulakis C.J. (1999). Neurotensin is a proinflammatory neuropeptide in colonic inflammation. Clin. Investig..

[22] Margolis K.G., Gershon M.D. (2009). Neuropeptides and inflammatory bowel disease. Curr. Opin. Gastroenterol..

[23] Piliponsky A.M., Chen C.C., Nishimura T., Metz M., Rios E.J., Dobner P.R., Wada E., Wada K., Zacharias S., Mohanasundaram U.M. (2008). Neurotensin increases mortality and mast cells reduce neurotensin levels in a mouse model of sepsis. Nat. Med..

[24] Barchetta I., Cimini F.A., Capoccia D., Bertoccini L., Ceccarelli V., Chiappetta C., Leonetti F., Di Cristofano C., Silecchia G., Orho-Melander M. (2018). Neurotensin Is a Lipid-Induced Gastrointestinal Peptide Associated with Visceral Adipose Tissue Inflammation in Obesity. Nutrients.

[25] Law I.K., Bakirtzi K., Polytarchou C., Oikonomopoulos A., Hommes D., Iliopoulos D., Pothoulakis C. (2015). Neurotensin--regulated miR-133α is involved in proinflammatory signalling in human colonic epithelial cells and in experimental colitis. Gut.

[26] Zhao D., Pothoulakis C. (2006). Effects of NT on gastrointestinal motility and secretion, and role in intestinal inflammation. Peptides.

[27] Brun P., Mastrotto C., Beggiao E., Stefani A., Barzon L., Sturniolo G.C., Palù G., Castagliuolo I. (2005). Neuropeptide neurotensin stimulates intestinal wound healing following chronic intestinal inflammation. Am. J. Physiol. Gastrointest. Liver Physiol..

[28] Akcan A., Muhtaroglu S., Akgun H., Akyildiz H., Kucuk C., Sozuer E., Yurci A., Yilmaz N. (2008). Ameliorative effects of bombesin and neurotensin on trinitrobenzene sulphonic acid-induced colitis, oxidative damage and apoptosis in rats. World J. Gastroenterol..

[29] Moura L.I., Dias A.M., Suesca E., Casadiegos S., Leal E.C., Fontanilla M.R., Carvalho L., de Sousa H.C., Carvalho E. (2014). Neurotensin-loaded collagen dressings reduce inflammation and improve wound healing in diabetic mice. Biochim. Biophys. Acta.

[30] da Silva L., Neves B.M., Moura L., Cruz M.T., Carvalho E. (2011). Neurotensin downregulates the pro-inflammatory properties of skin dendritic cells and increases epidermal growth factor expression. Biochim. Biophys. Acta.

[31] Pereira da Silva L., Miguel Neves B., Moura L., Cruz M.T., Carvalho E. (2014). Neurotensin decreases the proinflammatory status of human skin fibroblasts and increases epidermal growth factor expression. Int. J. Inflam..

[32] Van der Kleij H.P., Kraneveld A.D., van Houwelingen A.H., Kool M., Weitenberg A.C., Redegeld F.A., Nijkamp F.P. (2004). Murine model for non-IgE-mediated asthma. Inflammation.

[33] Kaczyńska K., Zając D., Wojciechowski P., Kogut E., Szereda-Przestaszewska M. (2018). Neuropeptides and breathing in health and disease. Pulm. Pharmacol. Ther..

[34] Robbins R.A., Nelson K.J., Gossman G.L., Rubinstein I. (1995). Neurotensin stimulates neutrophil adherence to bronchial epithelial cells in vitro. Life Sci..

[35] Aas P., Helle K.B. (1982). Neurotensin receptors in the rat bronchi. Regul. Pept..

[36] Djokic T.D., Dusser D.J., Borson D.B., Nadel J.A. (1989). Neutral endopeptidase modulates neurotensin-induced airway contraction. J. Appl. Physiol..

[37] Martin C.A., Gully D., Naline E., Advenier C. (1994). Neurotensin modulates cholinergic and noncholinergic neurotransmission in guinea-pig main bronchi in vitro. Neuropeptides.

[38] Tyler-McMahon B.M., Boules M., Richelson E. (2000). Neurotensin: Peptide for the next millennium. Regul. Pept..

[39] Gully D., Canton M., Boigegrain R., Jeanjean F., Molimard J.C., Poncelet M., Gueudet C., Heaulme M., Leyris R., Brouard A. (1993). Biochemical and pharmacological profile of a potent and selective nonpeptide antagonist of the neurotensin receptor. Proc. Natl. Acad. Sci. USA.

[40] Dubuc I., Costentin J., Terranova J.P., Barnouin M.C., Soubrié P., Le Fur G., Rostène W., Kitabgi P. (1994). The nonpeptide neurotensin antagonist, SR 48692, used as a tool to reveal putative neurotensin receptor subtypes. Br. J. Pharmacol..

[41] Schaeffer P., Laplace M.C., Bernat A., Prabonnaud V., Gully D., Lespy L., Herbert J.M. (1998). SR142948A is a potent antagonist of the cardiovascular effects of neurotensin. J. Cardiovasc. Pharmacol..

[42] Kaczyńska K., Szereda-Przestaszewska M. (2012). Cardio-respiratory effects of systemic neurotensin injection are mediated through activation of neurotensin NTS_1_ receptors. Eur. J. Pharmacol..

[43] Dobner P.R. (2005). Multitasking with neurotensin in the central nervous system. Cell. Mol. Life Sci..

[44] St-Gelais F., Jomphe C., Trudeau L.E. (2006). The role of neurotensin in central nervous system pathophysiology: What is the evidence?. J. Psychiatry Neurosci..

[45] Trinchieri G., Pflanz S., Kastelein R.A. (2003). The IL-12 family of heterodimeric cytokines: New players in the regulation of T cell responses. Immunity.

[46] Meyts I., Hellings P.W., Hens G., Vanaudenaerde B.M., Verbinnen B., Heremans H., Matthys P., Bullens D.M., Overbergh L., Mathieu C. (2006). IL-12 contributes to allergen-induced airway inflammation in experimental asthma. J. Immunol..

[47] Berkman N., Krishnan V.L., Gilbey T., Newton R., O’Connor B., Barnes P.J., Chung K.F. (1996). Expression of RANTES mRNA and protein in airways of patients with mild asthma. Am. J. Respir. Crit. Care Med..

[48] Zietkowski Z., Tomasiak M.M., Skiepko R., Bodzenta-Lukaszyk A. (2008). RANTES in exhaled breath condensate of stable and unstable asthma patients. Respir. Med..

[49] Agache I., Ciobanu C., Agache C., Anghel M. (2010). Increased serum IL-17 is an independent risk factor for severe asthma. Respir. Med..

[50] Bullens D.M., Truyen E., Coteur L., Dilissen E., Hellings P.W., Dupont L.J., Ceuppens J.L. (2006). IL-17 mRNA in sputum of asthmatic patients: Linking T cell driven inflammation and granulocytic influx?. Respir. Res..

[51] Dragon S., Rahman M.S., Yang J., Unruh H., Halayko A.J., Gounni A.S. (2007). IL-17 enhances IL-1beta-mediated CXCL-8 release from human airway smooth muscle cells. Am. J. Physiol. Lung Cell. Mol. Physiol..

[52] Fossiez F., Djossou O., Chomarat P., Flores-Romo L., Ait-Yahia S., Maat C., Pin J.J., Garrone P., Garcia E., Saeland S. (1996). T cell interleukin-17 induces stromal cells to produce proinflammatory and hematopoietic cytokines. J. Exp. Med..

[53] Izuhara K., Arima K., Kanaji S., Ohta S., Kanaji T. (2006). IL-13: A promising therapeutic target for bronchial asthma. Curr. Med. Chem..

[54] Wills-Karp M. (2004). Interleukin-13 in asthma pathogenesis. Immunol. Rev..

[55] Thomas P.S., Yates D.H., Barnes P.J. (1995). Tumor necrosis factor-alpha increases airway responsiveness and sputum neutrophilia in normal human subjects. Am. J. Respir. Crit. Care Med..

[56] Thomas P.S., Heywood G. (2002). Effects of inhaled tumour necrosis factor alpha in subjects with mild asthma. Thorax.

[57] Kim Y.S., Ko H.M., Kang N.I., Song C.H., Zhang X., Chung W.C., Kim J.H., Choi I.H., Park Y.M., Kim G.Y. (2007). Mast cells play a key role in the development of late airway hyperresponsiveness through TNF-alpha in a murine model of asthma. Eur. J. Immunol..

[58] Thomas P.S. (2001). Tumour necrosis factor-alpha: The role of this multifunctional cytokine in asthma. Immunol. Cell. Biol..

[59] Dai H., Korthuis R.J. (2011). Mast Cell Proteases and Inflammation. Drug Discov. Today Dis. Models.

[60] Kraneveld A.D., van der Kleij H.P., Kool M., van Houwelingen A.H., Weitenberg A.C., Redegeld F.A., Nijkamp F.P. (2002). Key role for mast cells in nonatopic asthma. J. Immunol..

[61] Sadowska-Woda I., Bieszczad-Bedrejczuk E. (2011). Rola stresu oksydacyjnego w patogenezie astmy oskrzelowej. Alergia Astma Immunologia.

[62] Abdelaziz R.R., Elmahdy M.K., Suddek G.M. (2018). Flavocoxid attenuates airway inflammation in ovalbumin-induced mouse asthma model. Chem. Biol. Interact..

[63] Muti A.D., Pârvu A.E., Muti L.A., Moldovan R., Mureşan A. (2016). Vitamin E effect in a rat model of toluene diisocyanate-induced asthma. Clujul. Med..

[64] Kogut E., Kaczyńska K., Lipkowski A., Kleczkowska P. (2017). Opioid-neurotensin hybrid influences pulmonary inflammatory process in murine model of non-atopic asthma. Eur. Respir. J..

[65] Tournoy K.G., Kips J.C., Schou C., Pauwels R.A. (2000). Airway eosinophilia is not arequirement for allergen-induced airway hyperresponsiveness. Clin. Exp. Allergy.

